# Music can effectively reduce pain perception in women rather than men

**DOI:** 10.12669/pjms.291.2947

**Published:** 2013

**Authors:** Sina Ghaffaripour, Hilda Mahmoudi, Mohammad Ali Sahmeddini, Abbas Alipour, Abdolhamid Chohedri

**Affiliations:** 1 Sina Ghaffaripour, MD, Shiraz Anesthesiology and Critical Care Medicine Research Center, Anesthesiology Department,; 2 Hilda Mahmoudi, MD, MPH, Shiraz University of Medical Sciences, Shiraz, Iran.; 3 Mohammad Ali Sahmeddini, MD, Shiraz Anesthesiology and Critical Care Medicine Research Center, Anesthesiology Department,; 4 Abbas Alipour, MD, Nutrition and Health School, Epidemiology Department, Shiraz University of Medical Sciences, Shiraz, Iran.; 5 Abdolhamid Chohedri, MD, Shiraz Anesthesiology and Critical Care Medicine Research Center, Anesthesiology Department, Shiraz University of Medical Sciences, Shiraz, Iran.

**Keywords:** Music, Pain tolerance, Pain rating, Cold pressor test

## Abstract

***Objective:*** Nowadays music is used to decrease pain and increase relaxation in clinical settings. It is hypothesized that music can affect women more easily than men. We assessed the effect of two types of music (Iranian folkloric and preferred music) on pain tolerance and pain rating in cold pressor test.

***Methodology:*** A consecutive sample of 50 healthy Iranian medical students was enrolled. They reported pain tolerance and pain rating in cold pressor test in three different musical conditions served as the outcome measures. The results were analyzed with repeated measurement analysis of variance.

***Result:*** Mean tolerance time was significantly higher in preferred music compared to Iranian folkloric music (F (1,48) =25.44, p=0.0001) and no music (F(1,48)=3.51, p=0.0001) conditions**.** There was a significant interaction when tolerance time in no music condition was compared to preferred music condition, regarding sex; Tolerance time increased more in females (F(1,48)=5.53, p=0.023). The results also indicated that pain ratings, regardless of sex, were different in three musical conditions (F(1.7,81.34)=15.37, p=0.0001).

***Conclusion:*** Music distracted attention from pain and Women can be impressed and distracted more easily by music.

## Introduction

 Ever since the creation of human being, varied necessities including physical and psychological needs have been accompanying him and he has been in need of some tools to fulfill these necessities and adjust himself with his environment.

 Art has been one of the first tools of human’s adjustment, which he has used to fulfill his desires for perfection and beauty, and also to ease the hardships and discomforts of life. Music is a form of art that conveys emotion, sentiment and perception without using language. Music affects mental and spiritual state and stimulates brain waves. Fast beat music makes brain alert and light music pacifies the brain and also affects the function of nervous system, softens the breath rate, reduces the heart rate, and keeps the body in complete peace. When body and mind are at peace, anxiety is reduced dramatically, inner processes are harmonized and immunity increased and all this proves that music can be a powerful therapy.

 Nowadays music is used to decrease pain, anxiety and increase relaxation in clinical settings. Some hospitals, take advantage of music to calm patients, accelerate the process of treatment and finally reduce the pain.^1^^,^^2^ Many studies have evaluated the effect of different types of music on pain. Regardless of type of music it seems that music has the positive effect on reducing pain.^3^

 It is hypothesized that music can affect women more easily than men because women are more emotional and sentimental. With this assumption, it is probable that music is more powerful pain distracter in women rather than in men. According to that hypothesis, we designed this experimental study to evaluate the effect of Iranian folkloric music and preferred music on men and women in pain tolerance and pain rating.

 One method for induction and assessment of pain is the cold pressor test (CPT). The cold pressor test has been recognized as a standard test to induce experimental pain since 1982. In this experimental study we used CPT to study the effect of Iranian traditional music and the preferred music on pain tolerance and pain intensity.

## Methodology

 After approval by Ethics Committee of Shiraz University of Medical Sciences, a consecutive sample of 50 healthy Iranian medical student volunteers (25 women, 25men) enlisted from Shiraz University of Medical Sciences was enrolled in study. They read and signed informed consent. Then they were instructed how to report pain tolerance and pain rating.

 Exclusion criteria contained presence of pain problem, vascular diseases, hypertension, cardiac diseases, Reynaud’s disease, skin disease and/or use of disease specific medications or hypnotic/psychotrop drugs.

 The cold tolerance test was used for this research and the procedure was as follows: Every single participant was tested in a quiet environment. Participants had to immerse their hands to their wrists in a container that contained water and ice with a temperature between 0°-4° centigrade (The temperature was monitored by a digital thermometer). The palm of their hands could not touch the container. This test was done for each individual in three different conditions and in proper intervals of at least 60 minutes so that the endorphins would not be effective any more.

 The standard duration of this test was between 0-180 seconds, which varied depending on the individual’s tolerance. Pain intensity was measured by asking each person every 15 seconds to evaluate the intensity of pain he or she felt on a diagram graduated from 0 to 10 (Numerical pain intensity). Before the test, it was explained to each person that 0 means no pain and 10 means intolerable pain. These numbers were recorded by an examiner every 15 seconds. The test was done for each participant between 5-7 pm in these three conditions:

1. While listening to no music in a quiet and noiseless environment, from the beginning of the test to the end.

2. While listening to one specific Iranian folkloric music which was the same for all participants, the music was played by MP3 player and through headphones, from the beginning of the test to the end.

3. While listening to preferred music that was selected by the person her/himself and played by MP3 player and through headphones, from the beginning of the test to the end.

 These three conditions were counter balanced to avoid confounding of musical condition effect with those of time/habituation. A research assistant who was blinded to the main purpose of the study worked with the participants during the study. It was the same person for every participant.

***Data analysis tests and statistical procedures: ***Tolerance time and pain rating served as the outcome measures. The results were analyzed with repeated measurement analysis of variance, with gender as a between subject factor and musical condition as a within subject factor.

 Because tolerance time values did not follow normal distribution in both musical conditions, log transformation was done. Thus geometric mean ±standard deviation (SD) was calculated (exponential of mean reported in statistical analysis by software) besides arithmetic mean±SD. Assumption of sphericity was violated in Muchly test (p=0.01) and therefore after adjustment of degree of freedom by correlation coefficient of Greenhouse – Gisser (ε=0.848) and Huynh- Feldt (ε=0.895), average p-value of those assumptions was reported.

 Average scores values in all musical conditions followed normal distribution. Thus arithmetic mean ± S.D was reported. Again assumption of sphericity was not met in Muchly test (p=0.009) and therefore after adjustment of degree of freedom by correlation coefficient of Greenhouse – Gisser (ε=0.847) and Huynh- Feldt (ε=0.893), average p-value of those assumptions was reported.

 SPSS program (version 13, Chicago, IL, USA) was used for statistical analysis. Two sided P value less than 0.05 considered significant.

**Table-I:** Mean tolerance time in three musical conditions

*Musical condition*	*No music* ^1^		*Iranian folkloric music* ^1^		*Preferred music* ^1^	
Total	66.3 ± 40.33	55.66 ± 1.86	77.1 ± 45.36	64.97 ± 1.84	84.9 ± 47	72.24 ± 1.83
Female	56.4 ± 38.07	46.55 ± 1.88	66.6 ± 40.87	56.69 ± 1.78	76.8 ± 46.1	65.3 ± 1.8
Male	76.2 ± 40.83	65.96 ± 1.77	87.6 ± 47.96	74.47 ± 1.85	93 ± 47.43	79.9 ± 1.84

^1^first line is mean ± standard deviation and second line is geometric mean ± standard deviation.

## Results

 In this study 25 women (mean age: 20.64 ±1.8) and 25 men (mean age: 20.52±0.2) were enrolled. Ages ranged from 18 to 24 in both groups and the means were not statistically different (CI: - 0.98±1.22) with respect to sex.

 The results indicated that mean tolerance times, collapsing across sex, were significantly different in three musical conditions (F(1.7,81.54)=48.56, p=0.0001). Mean tolerance time was significantly higher in preferred music compared to Iranian folkloric music (F(1,48)=25.44, p=0.0001) and no music (F(1,48)=3.51, p=0.0001) conditions. Similarly, mean tolerance time was higher in Iranian music compared to no music condition (F(1,48)=1.27, p=0.0001) (Table-I).

**Fig.1:**
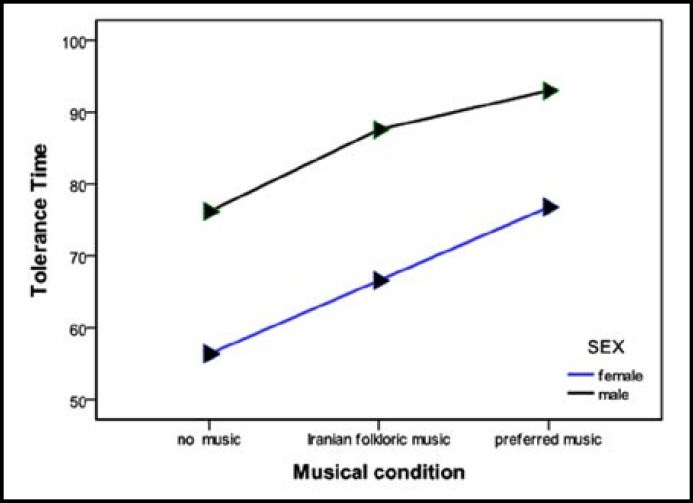
Mean tolerance time in three musical conditions divided by sex

 There was a significant interaction effect between tolerance time in musical conditions and sex (F(1.7,81.54)=3.65, p=0.037). This revealed significant interaction when tolerance time in no music condition was compared to preferred music condition, regarding sex; Tolerance time increased more in females. (F(1,48)=5.53, p=0.023). However no interaction was observed comparing tolerance time in Iranian music condition to no music (F(1,48)=1.81,p=0.185) and preferred music(F(1,48)=2.84, p=0.09) conditions with respect to sex. (Fig.1)

 The results also indicated that pain ratings, regardless of sex, were different in three musical conditions (F(1.7,81.34)=15.37, p=0.0001). Pain ratings were higher in no music condition compared to preferred music (F(1,48)=22.44, p=0.0001) and Iranian music (F(1.48)=5.85, p=0.019) conditions. Moreover, pain rating in Iranian music condition was higher compared to preferred music condition (F(1,48)=15.31, p=0.0001). (Table-II)

**Table-II:** Average scores of pain rating in three musical conditions

	*Without music* ^1^	*Iranian traditional music* ^1^	*Preferred music* ^1^
Overall	7.6 ± 1.39	7.4 ± 1.31	7.15 ± 1.36
Female	7.1 ± 1.36	6.88 ± 1.2	6.68 ± 1.32
male	8.19 ± 1.16	7.92 ± 1.22	7.61 ± 1.24

^1^Mean ± SD

**Fig.2:**
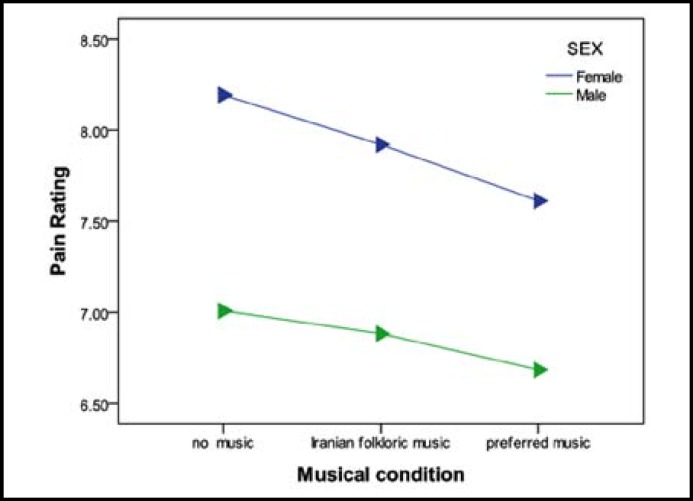
Average scores of pain rating in three musical conditions divided by sex.

 Finally, no significant interaction effect was revealed between musical conditions and sex (F(1.7,81.34)=1.24,p=0.29),in other words there was no significant sex difference in pain ratings. (Fig.2)

## Discussion

 Our study has shown that music had a distractive effect in pain feeling because attention converts from pain stimuli to a lovely and enjoyable incentive. This experimental study supports many other studies which noted that music can reduce pain in clinical settings such as postoperative pains^4^^-^^7^, post invasive procedures pain^8^ or pain experiencing by post anesthetics care units patients.^9^^,^^10^ Moreover, it has also been shown that music can be used as a therapeutic modality in treatment of chronic pains and diseases.^11^ However some other investigators have reported that music does not have any significant effect in reducing pain experience.^12^^,^^13^

 In present study we concluded that preferred music was more powerful in distraction attention from pain. Although Iranian folkloric music was still effective in increasing pain tolerance and decreasing pain rating comparing to no music condition. On one hand, Preferred music seems to have emotional and memorial engagements with individuals’ fond memories, thus it is more powerful in diverting individuals’ attention from pain.^14^ On the other hand, most of Iranian folkloric music like the piece that we had chosen is slow-beat and low frequency music. These kinds of music may raise lower levels of energy to increase pain tolerance. In present survey, studied group were young and their ages were between 18 to 24 years old. Most of them chose fast beat music as a preferred music that could raise person’s mood and attract their attention more easily.

 Mitchel and colleagues noted that preferred music can increase pain tolerance more effectively, which is in agreement with us even though unlike our findings. They have reported that pain rating did not differ significantly.^14^ Some studies have indicated that women reported more pain and had lower pain tolerance compared to men,^15^^-^^18^ while some studies have reported that tolerance is higher meanwhile rating is lower in males.^19^^-^^22^

 In some other studies there was no difference between men and women in relation to pain rating however pain tolerance was higher in men.^14^^,^^23^^,^^24^ Fillingim and coworkers in their systematic review revealed that in most studies females are more sensitive to pain especially in pain tolerance and rating.^25^ Similarly, we found that, tolerance time in women was lower when there was no music, but the interesting point was that when these women were listening to their favorite music, they tolerated the cold water for longer duration. This finding was significantly more dominant in women than in men. This unique finding means that women are more responsive to music specially preferred music than men. Women are more sensitive and sentimental and can be impressed and distracted more easily by music. This finding supports the theory that music act as a distracter to reduce pain which is commonly accepted.^3^ We conclude that music is an effective pain reducer in an experimental situation and many other studies in clinical condition support our results. However this new idea that music can be more useful for women should be confirmed by more experimental and clinical researches.
